# Drivers and variability of CO_2_:O_2_ saturation along a gradient from boreal to Arctic lakes

**DOI:** 10.1038/s41598-022-23705-9

**Published:** 2022-11-08

**Authors:** Lina Allesson, Nicolas Valiente, Peter Dörsch, Tom Andersen, Alexander Eiler, Dag O. Hessen

**Affiliations:** 1 Section for Aquatic Biology and Toxicology, Department of Biosciences, Centre for Biogeochemistry in the Anthropocene, University of Oslo, 0316 Oslo, Norway; 2 Division of Terrestrial Ecosystem Research, Centre for Microbiology and Environmental Systems Science, University of Vienna, 1030 Vienna, Austria; 3 Faculty of Environmental Sciences and Natural Resource Management, Norwegian University of Life Sciences, 1432 Ås, Norway

**Keywords:** Biophysics, Biogeochemistry, Environmental sciences, Limnology

## Abstract

Lakes are significant players for the global climate since they sequester terrestrially derived dissolved organic carbon (DOC), and emit greenhouse gases like CO_2_ to the atmosphere. However, the differences in environmental drivers of CO_2_ concentrations are not well constrained along latitudinal and thus climate gradients. Our aim here is to provide a better understanding of net heterotrophy and gas balance at the catchment scale in a set of boreal, sub-Arctic and high-Arctic lakes. We assessed water chemistry and concentrations of dissolved O_2_ and CO_2_, as well as the CO_2_:O_2_ ratio in three groups of lakes separated by steps of approximately 10 degrees latitude in South-Eastern Norway (near 60° N), sub-Arctic lakes in the northernmost part of the Norwegian mainland (near 70° N) and high-Arctic lakes on Svalbard (near 80° N). Across all regions, CO_2_ saturation levels varied more (6–1374%) than O_2_ saturation levels (85–148%) and hence CO_2_ saturation governed the CO_2_:O_2_ ratio. The boreal lakes were generally undersaturated with O_2_, while the sub-Arctic and high-Arctic lakes ranged from O_2_ saturated to oversaturated. Regardless of location, the majority of the lakes were CO_2_ supersaturated. In the boreal lakes the CO_2_:O_2_ ratio was mainly related to DOC concentration, in contrast to the sub-Arctic and high-Arctic localities, where conductivity was the major statistical determinant. While the southern part is dominated by granitic and metamorphic bedrock, the sub-Arctic sites are scattered across a range of granitic to sedimentary bed rocks, and the majority of the high-Arctic lakes are situated on limestone, resulting in contrasting lake alkalinities between the regions. DOC dependency of the CO_2_:O_2_ ratio in the boreal region together with low alkalinity suggests that in-lake heterotrophic respiration was a major source of lake CO_2_. Contrastingly, the conductivity dependency indicates that CO_2_ saturation in the sub-Arctic and high-Arctic lakes was to a large part explained by DIC input from catchment respiration and carbonate weathering.

## Introduction

Oxygen (O_2_) and carbon dioxide (CO_2_) are key gases for life on Earth. Their ratio is mainly determined by the balance between heterotrophic and autotrophic processes in the biosphere. Under net autotrophic conditions, CO_2_ is sequestrated in both terrestrial and aquatic ecosystems, thus decreasing atmospheric CO_2_ concentrations and greenhouse effects on global climate. Lakes, and notably boreal lakes, are key players in this context since they convert a significant part of terrestrially derived organic carbon to CO_2_ and CH_4_^1,2^. A large share of CH_4_ may also be converted to CO_2_ by methanotrophs in the water column^3^.

Simultaneous and complementary biological processes thus drive variations in O_2_ and CO_2_ concentrations^4,5^ and this coupling has led to the assumption that O_2_ and CO_2_ can be used interchangeably when studying metabolism of aquatic ecosystems. O_2_ is advantageous over CO_2_ because it can be measured directly in the aqueous phase without gas extraction. Therefore, indirect assessments of CO_2_ based on dissolved O_2_ are often favored over direct CO_2_ measurements^6,7^. However, combined CO_2_ and O_2_ measurements have shown decoupling over time^8–10^. This may in part be explained by decoupling of production and consumption of these gases across the aquatic-terrestrial interface and thus reflect the catchment type of the aquatic ecosystem^9^. Other explanations for the deviations in CO_2_:O_2_ coupling could be inputs of CO_2_-rich water and anaerobic CO_2_ cycling^11^. Simultaneous measurements of CO_2_ and O_2_ concentrations thus increase the understanding of lake ecosystem functioning. Ratios of CO_2_:O_2_ may in turn give a better indication of lake net heterotrophy than CO_2_ or O_2_ alone, because production and decomposition affect the nominator and the denominator of the ratio with opposite signs. Thus we would expect a stronger response to increased heterotrophy in the CO_2_:O_2_ ratio than in CO_2_ alone. It will also provide insight in spatial drivers of CO_2_ emissions that could allow a space-for-time approach to address future changes in e.g. climate, hydrology, forest cover and other catchment properties.

Biological oxidation of dissolved organic matter (DOM) is a main source of autochthonous aquatic CO_2_^12–14^ while photochemical oxidation plays a smaller role^14,15^. Other sources of CO_2_ may be allochthonous, e.g. from catchment processes like root exudation or supersaturated groundwater (Raymond et al., 2013). Depending on the pH and buffering capacity of the water, inorganic carbon is either present as CO_2_ or reacts with water forming bicarbonate or carbonate. Lake chemistry and notably pH thus plays a key role in determining the CO_2_ concentration in the water. Similarly, elevated temperatures will reduce concentrations while not necessarily the level of saturation or the CO_2_:O_2_ ratio, as the dissolution of both gases depends similarly on temperature.

The Boreal zone is characterized by extensive land–water interfaces. Forests with large above- and below-ground C-pools as well as abundant bogs and wetlands, export terrestrial DOM and dissolved inorganic carbon (DIC) to waters, making boreal aquatic ecosystems an especially important component of the global carbon cycle^2^. In certain areas also agricultural areas may be important sources of organic C. Climate change, changes in land use, most notably afforestation, and the recovery from acidification^16^ have led to increased export of terrestrial DOM and a shift in water color towards brown in many boreal freshwaters^17,18^. This “browning” may affect lake metabolism in various ways. Terrestrially derived DOM provides energy and nutrients to heterotrophs, stimulating bacterial metabolism and CO_2_ production^19,20^. Nutrients associated to DOM may also benefit autotrophs. However, the beneficial effect of enhanced nutrient loadings on autotrophs is overridden by enhanced light attenuation inhibiting primary production as DOM concentration increases^20,21^. This trade-off between positive and negative effects of increasing DOM inputs on photosynthesis may yield a unimodal response in primary production with a maximum around 5–10 mg DOC l^−1^^22,23^.

In contrast to boreal lakes, Arctic lakes have generally sparsely forested, or unforested catchments with less developed soils, yet there is a gradient from sub-Arctic to high-Arctic sites. Still they are generally supersaturated with and net emitters of CO_2_ to the atmosphere^24^. Lake abundancy is higher in the Arctic than in any other region of the world with high complexity in lake type ranging from clear, pristine, mountain lakes to brown, DOM rich thermokarst lakes formed by thawing permafrost^25–27^. Climate warming in the Arctic has led to increased biomass and production of terrestrial plants and schrubs, so called Arctic greening, leading to increased loads of DOM to Arctic lakes^28–30^. At the same time, thawing permafrost results in inputs of old organic carbon to Arctic lakes that may undergo microbial oxidation^31–33^. Increased DOM loadings from thawing permafrost are thus expected to result in enhanced CO_2_ production and emission from Arctic lakes^30,34,35^.

Autochthonous (in-lake) production is however not the only source of DIC. Lateral flux of inorganic carbon produced in the catchment may account for a sizeable share of lake CO_2_, especially in small lakes with short retention times and long ice-free seasons^36^. In-lake DOM mineralization together with catchment derived (allochthonous) DIC inputs make most lakes worldwide supersaturated with- and net emitters of CO_2_ to the atmosphere^1,37^.There are conflicting reports of whether CO_2_ produced in aquatic environments via DOM mineralization or exported from terrestrial environments is the main regulator of lake CO_2_ flux^36,38–40^. Some of these contradictions likely depend on climate, local hydrology, catchment slopes, water retention time, and not the least catchment properties like lake size or fraction and type of forest, bogs and wetlands^41,42^.

In this study we assess the patterns and drivers of CO_2_:O_2_ ratios in lakes along a latitudinal gradient to see if these drivers vary between the distinctly different sets of lakes. More specifically we aim to gain understanding of lake CO_2_ sources, whether they are dominated by in-lake processes or by allochthonous inputs and whether there is a latitudinal difference in drivers of lake CO_2_:O_2_ ratio, notably related to forested or unforested catchments. To do so, we couple surface CO_2_ and O_2_ concentrations in 103 Norwegian lakes to environmental variables along a geographical gradient ranging from the boreal zone in southern Norway (58° N) through sub-Arctic northern Norway (69 ^o^ N) to the high Arctic at Svalbard (79° N). The gradient reflects different catchment properties varying from dense spruce forest, via open birch forest to totally unforested catchments with shallow soils in the high Arctic.

This wide spatial gradient across climatic regions and catchment properties should provide insights relevant to larger parts of both the boreal and the arctic biome, yet with the proviso that there clearly may be pronounced regional differences within this vast area.

## Materials and methods

### Study lakes

**Figure 1:**
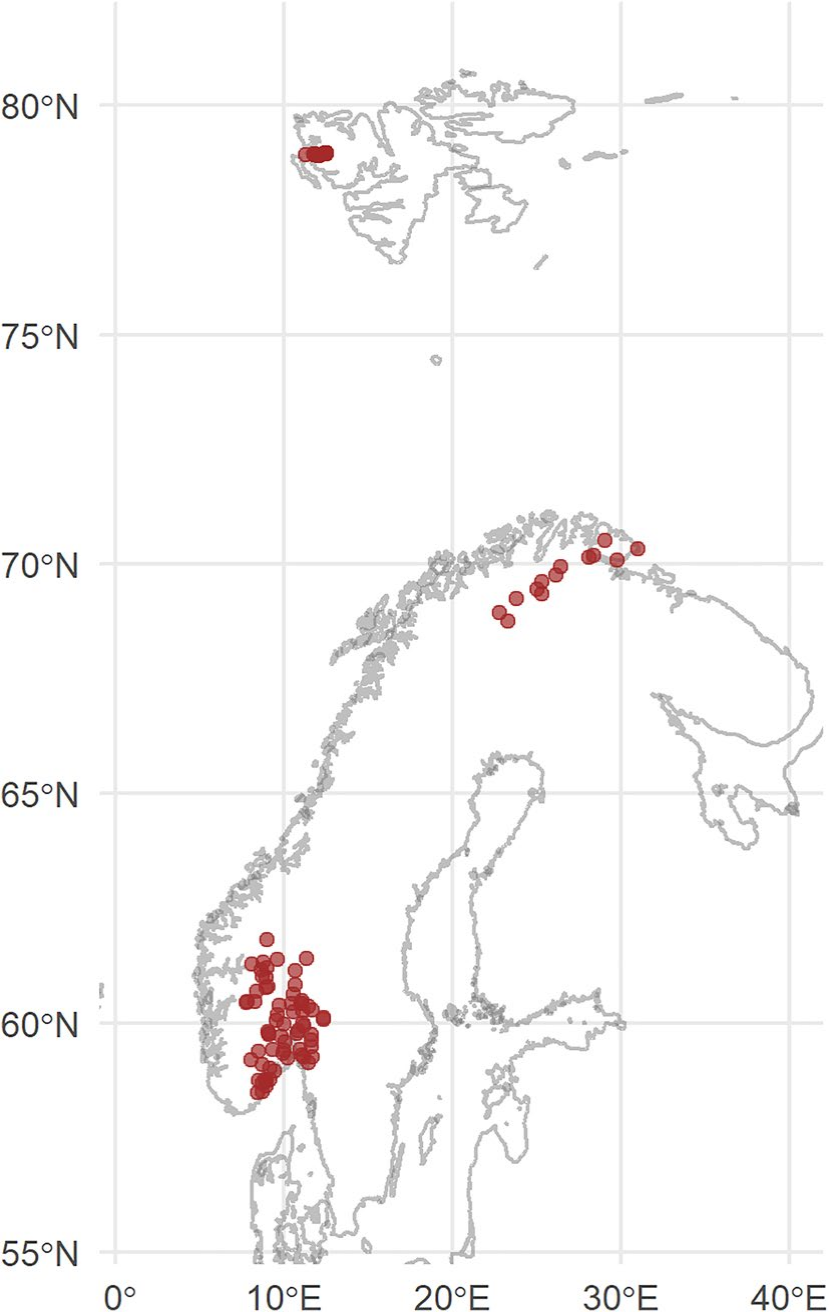
Map of the site locations. The map is generated by R (version 3.4.1): rnaturalearth (https://cran.r-project.org/web/packages/rnaturalearth/README.html) kombined with sf (https://cran.r-project.org/web/packages/sf/index.html).

During fall 2019, 73 lakes in South-Eastern Norway (Boreal) and 22 Arctic lakes on Svalbard (high-Arctic) were sampled and a number of water chemistry parameters and dissolved gases were measured. In fall 2020, additionally 14 sub-Arctic lakes in the Finnmark county (Northern Norway; sub-Arctic) were sampled (Fig. 1). The boreal, southern cluster of lakes is within the coniferous forest zone but covers locations differing in size and altitude. The sub-Arctic lakes have sparsely forested catchments with birch, less topographic variation and larger areas of bogs and wetlands, while the high Arctic sites are treeless and also with very sparsely developed soils. Granitic and metamorphic bedrock dominate in the southern zone, yielding low alkalinity lakes (Fig. S1). The catchments of these lakes are also characterized by well-developed soils. The sub-Arctic lakes are situated on granitic to sedimentary rocks (slate) giving rise to a wide range in alkalinity. The sub-Arctic lakes were chosen to span a geographical gradient from the southern inland to the costal northeast, reflecting a gradient in biome domination from taiga to Arctic tundra. A high proportion of the high-Arctic lakes are situated on limestone bedrock, with elevated alkalinity (Fig. S1). The high-Arctic lakes are mostly small with catchments devoid of soil and vegetation beyond scrubs and mosses, covering a gradient from rocky terrain from glacier fronts to shore sites, the latter influenced by birds and with some vegetation^43^.

### Sampling and field sample preparations

Sampling was performed at, or shortly after, fall overturn, to secure a maximum vertical homogenization of the water masses and gases. Since onset of fall differs with latitude, the samplings were performed during October, September and August for the three clusters of lakes (Boreal, sub-Arctic and high-Arctic, respectively), reflecting their geographical position from south to north. This procedure was based on the procedures adopted in the national surveys on water chemistry in Northern Europe, initiated to address the impact of acidification on lake water chemistry^44^. The rationale for this is primarily that following mixing, the water masses are homogenized and thus the sampled water volume should be more representative than a surface sample during stratification. No doubt the dissolved gases may differ with season and depth. Potentially autumn samples could yield higher CO_2_:O_2_ ratios than summer samples (reduced photosynthesis relative to respiration), but since a full seasonal and vertical inventory of all lakes for obvious reasons could not be performed, this post-overturn sampling would serve as an integrated fingerprint of lake metabolism. Since only few localities were accessible from road, sampling from boat was problematic. Hence the sampling was performed from the shore using a designed 4 m sampling rod with a holder and beaker at the end. All localities were collected during daytime (11 a.m to 2 pm) at three different sites, preferably representing different substratum and shoreside properties, yet in line with the designed, survey of North European lakes, near the outlet wherever this could be identified, and the three sites were pooled into one sample. Again, the benefit of sampling after fall overturn is that local impacts would, if not cancelled out, at least be minimized by mixing. Also, most sites were rather small headwater lakes, and the high Arctic sites mostly ponds, meaning that sampling 4 m from the shore should be representative of the mixed water masses.

At the sampling sites, in situ measurements of temperature (T), pH and electrical conductivity (EC) were taken using a portable multimeter (Hach HQ40D). Samples were processed in situ for further analysis of lake chemistry: 50 ml of unfiltered water was taken for the analysis of total organic carbon (TOC), total nitrogen (TN) and total phosphorus (TP). For analysis of DOC, dissolved nitrogen (DN) and phosphorus (DP), surface water from the bucket was filtered in the field through Sterivex 0.22 µm pore size filters (Millipore). All samples for water analysis were kept cool (4 °C) until back from the field (the same evening), where they were frozen at − 20 °C until further analysis at the University of Oslo, generally within 2 to 4 weeks. Dissolved CO_2_, O_2_ and argon (Ar; all sites except Svalbard) were analyzed using the headspace method after acidification^45^. The samples were prepared in the field by filling 30 ml water in a syringe, creating an in-situ headspace with air (20 ml) and adding 0.6 ml of 3% HCl (≈ 1 M). Syringes were closed and equilibrated by shaking the syringes for 3 min. In situ lake temperature was used as equilibration temperature and caution was exercised to not warm the syringes during shaking. The equilibrated headspace gas was then injected into He-washed and pre-evacuated glass vials crimp sealed with butyl rubber septa, and then kept at room temperature until analysis by gas chromatography.

### Laboratory analysis

TOC and DOC were measured by infrared CO_2_ detection after catalytic high temperature combustion (Shimadzu TOC-VWP analyser). TP was measured on a SEAL AA3 HR AutoAnalyzer (SEAL Analytical Inc., Mequon, Wisconsin 53092, USA) as phosphate after wet oxidation with peroxodisulfate. TN was measured on unfiltered samples by detecting nitrogen monoxide by chemiluminescence using a TNM-1 unit attached to the Shimadzu TOC-VWP analyser. Concentrations of CO_2,_ O_2_ and Ar were determined by automated gas chromatography (GC) analysis with He back-flushing as described by Yang et al.^13^. The level of saturation of each gas relative to equilibrium with ambient air was calculated from measured concentrations using Henry’s law with temperature-dependent solubility constants. In the sites where Ar was measured, O_2_ was normalized to Ar saturation for a more stable and accurate estimate of O_2_ saturation^46^.

### Statistical analysis

All data analysis was performed using the open-source software R version 3.4.1^47^. We used Spearman’s rank correlation as a measure of association between continuous variables. To calculate DIC from pH and CO_2_ at the in situ temperature, we used the AquaEnv package^48^. For the statistical modelling, we used the mgcv package^49^ to fit generalized additive models (gam) of the gaussian family to the dependent variables. To test the dependency of CO_2_:O_2_ ratio and O_2_ saturation, we used a gam model with smoothers on each of the explanatory variables DOC (mg l^−1^), TP (µg l^−1^), TN (mg l^−1^), and conductivity (EC; µS cm^−1^). Predictive variable selection was done by applying additional shrinkage on the null space of the penalty with the select = TRUE argument in the mgcv::gam function, as recommended by Marra and Wood^50^. The resulting models have all smoothers that are not necessary for the fit as close to zero as possible.

## Results

**Figure 2:**
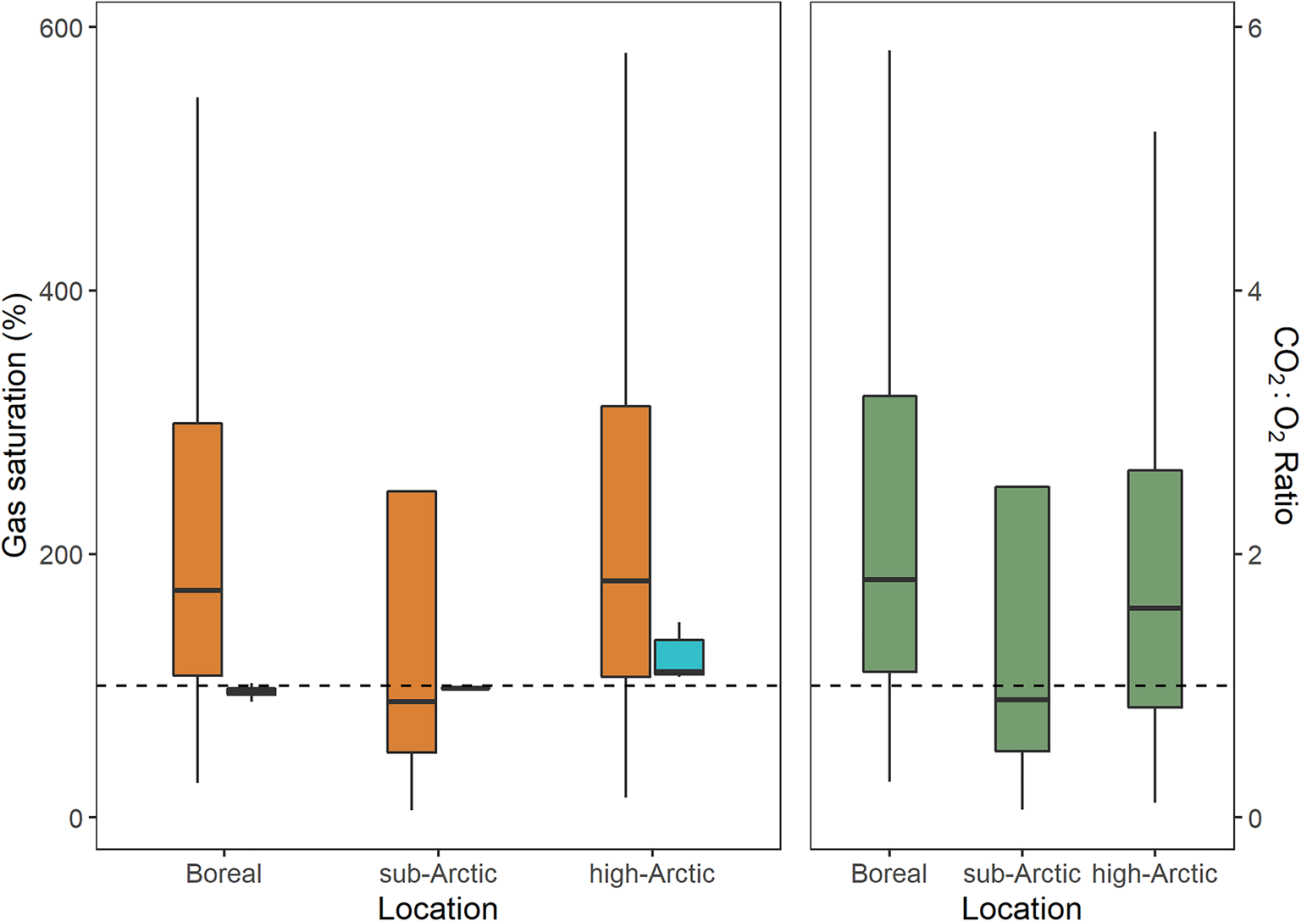
Box plot of lake CO_2_ saturation (red), O_2_ saturation (blue), and CO_2_:O_2_ ratio (green) in the different geographical regions. Dashed line represents the 100% saturation (to the left) and a CO_2_:O_2_ ratio of 1 (to the right).

Saturation levels of O_2_ varied with geographical location (Fig. 2). By contrast, CO_2_ saturation covered a much wider range with both undersaturated and supersaturated lakes in the sub-Arctic and saturation or supersaturation in the boreal and the high-Arctic lakes (Table 1; Fig. 2).

**Table 1:** Statistics of the lake chemistry variables used in the analyses.

	Boreal	Sub-Arctic	High-Arctic
CO_2_ saturation range (%; median; average)	26–1374 (175; 260)	6–1218 (88; 262)	15–1121 (180; 245)
O_2_ saturation range (%; median; average)	85–102 (96; 95)	95–99 (98; 98)	107–148 (111; 120)
DOC range (mg l^−1^; median; average)	1.5–20.7 (7.3; 7.7)	2.8–20.9 (6.6; 8)	1–14 (4; 4.9)
TP range (µg l^−1^; median; average)	3–49 (9; 12)	3–11 (6; 6.5)	2–19 (3; 6.2)^a^
TN range (mg l^−1^; median; average)	0.05–1.07 (0.27; 0.33)	0.16–0.79 (0.27; 0.35)	0.003–0.8 (0.08; 0.2)
Conductivity range (µS cm^−1^; median; average)	6–243 (24.5; 42)	11–74.8 (40; 44.4)	16.5–1410 (212; 269)

^a^12 out of 22 lakes with concentrations below detection limit.

The majority of the boreal lakes were supersaturated with CO_2_ and undersaturated with O_2,_ with a weak but significantly negative correlation between CO_2_ and O_2_ saturation levels (ρ = − 0.42, p < 0.001). The sub-Arctic and high-Arctic lakes, which were all saturated or supersaturated with O_2_, showed deviating patterns of CO_2_ saturation with no significant correlation between CO_2_ and O_2_ saturation levels (Fig. S2). Across all three latitudinal zones, lake O_2_ saturation levels showed lower variation than CO_2_ saturation levels. Hence, the variation in CO_2_:O_2_ ratio was mainly governed by CO_2_ saturation (Fig. 2).

### General patterns among nutrients and conductivity

While high Arctic lakes had lower DOC concentrations than boreal lakes on the mainland, there were no major differences in DOC concentrations among the boreal and the sub-Arctic mainland lakes (Table 1). In the boreal lakes, DOC concentrations correlated positively with TN and total conductivity (ρ = 0.48 and ρ = 0.45, respectively; Fig. S3). The relation between DOC and TP was also positive but weak. Conductivity ranged between 6 and 243 µS cm^−1^ with a median value at 24 µS cm^−1^ (Table 1) and correlated best with TN (ρ = 0.72; Fig. S3).

Similar to the boreal lakes, DOC was positively correlated to TN in both the sub-Arctic and in the high-Arctic lakes (ρ = 0.42 and ρ = 0.65, respectively). However, there was no significant relation between DOC and conductivity in the high-Arctic while in the sub-Arctic, the relation was weak but negative (Fig. S3). DOC and pH correlated positively in the high-Artic (ρ = 0.65) and in the sub-Arctic lakes there was a positive correlation between conductivity and pH (ρ = 0.59). TP was generally lower in the high-Arctic than in the boreal and sub-Arctic lakes and 12 of the 22 lakes in the high-Arctic had TP concentrations below detection limit. Conductivity in the high-Artic lakes was generally higher and had a wider range than in the boreal lakes (Table 1).

In all lakes, there was a strong positive correlation between conductivity and DIC concentrations (Fig. S3). In the boreal lakes, DIC concentrations were generally low. Contrastingly, in the Arctic the relationship between DOC and DIC was either negative (sub-Arctic) or non-existing (high-Arctic) and thus inorganic compounds are the main sources of alkalinity in the arctic lakes. Including all lakes in the full model with DOC, TP, TN, and conductivity as independent variables, the model explained 42% of the deviance, with conductivity, TP and DOC concentration as significant predictors (Table 2). Since the variables predicting the CO_2_:O_2_ ratio differed with geographic location, the models were also run for each zone separately.

**Table 2:** Model outputs of the general additive models (CO_2_:O_2_ ~ s(DOC) + s(TP) + s(TN) + s(cond)) for the different regions.

	Variance explained (%)	R^2^_adj_	Significant predictors
All lakes	42	0.32	DOC*, TP*, cond***
Boreal lakes	73	0.63	DOC***, TP**, TN*, cond**
Sub-Arctic lakes	75	0.69	TN**, cond***
High-Arctic lakes	94	0.9	DOC*, TN**, cond***

Significance codes: ***p < 0.001, **p < 0.01, *p < 0.05, ^non-sig^p > 0.05.

### Boreal zone

**Figure 3:**
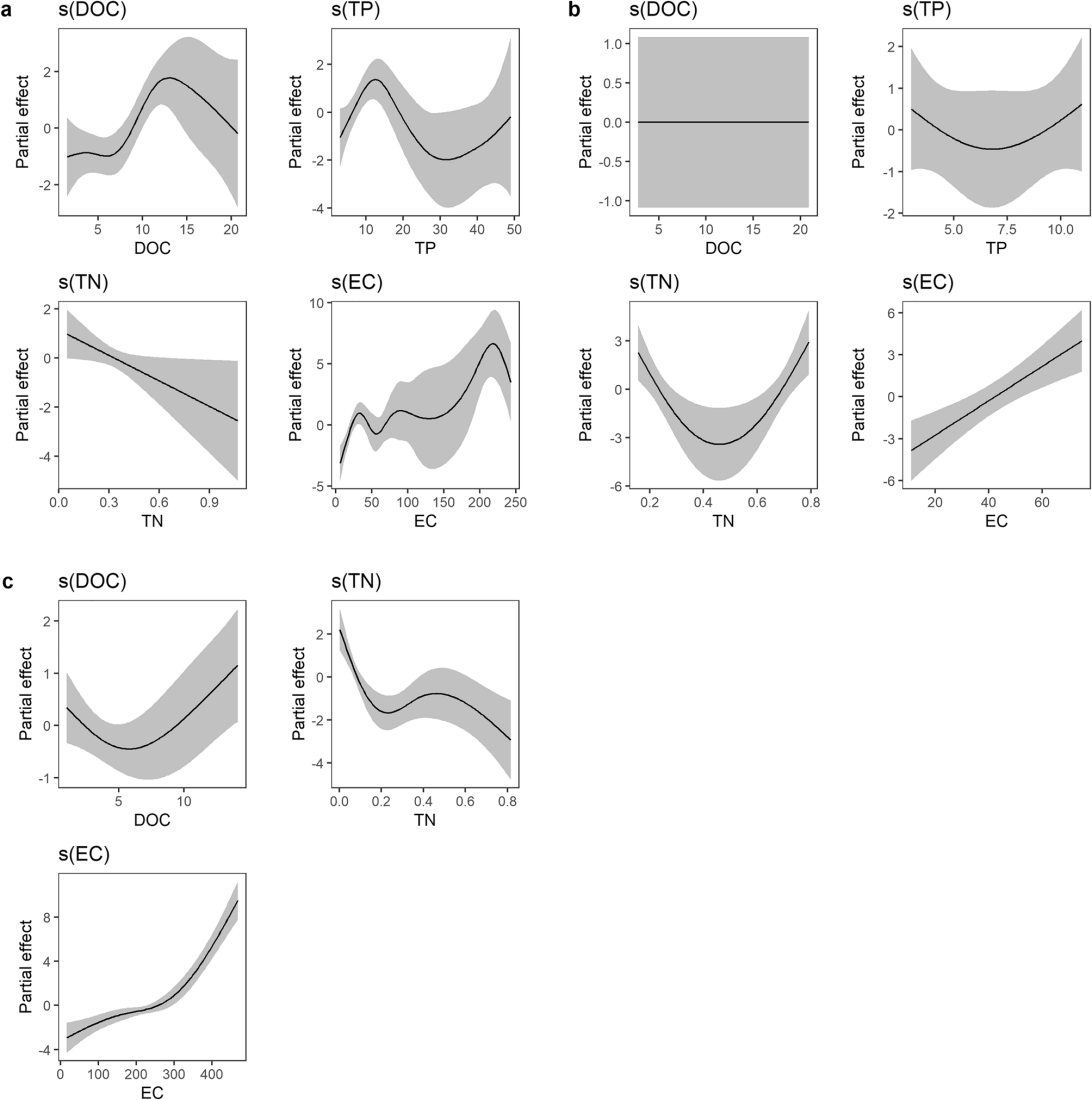
Result plots of the generalized additive models (gams) predicting CO_2_:O_2_ saturation ratio for the three regions. In the Boreal lakes (**a**), the best predictor was DOC concentration followed by conductivity. In both the sub-Arctic (**b**) and high-Arctic (**c**), conductivity was the best predictor for CO_2_:O_2_ saturation ratio.

As stated, the CO_2_ and O_2_ saturation were negatively related with each other in the boreal lakes. A model with DOC as the only explanatory variable explained 35% of the observed deviance. The full model, including TN, TP, and conductivity, improved the explained deviance to 73% (Table 2; Fig. 3a). DOC and conductivity were the strongest predictors, both having positive effects on the CO_2_:O_2_ ratio. O_2_ saturation in the boreal lakes was negatively related to DOC concentrations and positively related to TN (Fig. S3).

### High-and sub-Arctic lakes

CO_2_:O_2_ ratios in the Arctic lakes were primarily associated with conductivity (positive) and TN (negative) with the models explaining 75% and 98% of the deviance for the sub-Arctic and the high-Arctic, respectively (Table 2; Fig. 3b,c). In the high-Arctic, DOC was a weak (p = 0.04) and mainly negative predictor of CO_2_:O_2_ ratio while TP was below detection limit in most lakes and excluded from the analysis. In the sub-Arctic, neither DOC nor TP were significant predictors of CO_2_:O_2_ ratio.

All lakes in the Arctic were saturated with O_2_. In the sub-Arctic, O_2_ saturation was at 100% in all lakes without variation among lakes and, hence, no correlation was found between O_2_ saturation and any other variable. In the high-Arctic, O_2_ saturation spanned a wider range from saturation to oversaturation. The only variable that correlated with O_2_ saturation was conductivity (ρ = − 0.48). Modelling the CO_2_:O_2_ ratio in the high-Arctic lakes with conductivity as the only independent variable explained 69% of the deviance. In the sub-Arctic, 70% of the deviance was explained with conductivity as the only explanatory variable.

## Discussion

### O_2_ and CO_2_ saturation

Our study revealed striking differences between boreal lakes on the one hand, and sub-Arctic and high-Arctic sites on the other. The boreal lakes (n = 73) all had catchments dominated by coniferous forests, primarily spruce and pine and the terrestrial C-fixation by forests enrich also lake water with DOC^17,18,51^, which in turn promote the biogenic production of CO_2_ from bacterial respiration^1,2,14,38^. The positive relationship between CO_2_ saturation and DOC concentration indicates in-lake CO_2_ production by microbial DOC mineralization. Still CO_2_ saturation can be affected by retention time, as under low retention times DOC and CO_2_ flushed in from the catchment into systems might increase saturation. Short retention times are also associated with inputs of highly reactive DOC^52^, yielding enhanced CO_2_ production rates. As such the positive relationship between CO_2_ saturation and DOC concentration needs to be interpreted with caution in systems with low retention time as it is likely impacted by complex interactions of allochthonous and autochthonous processes.

Besides biological processes and air–water exchange, lateral input via groundwater and surface run-off contribute to lake CO_2_^53,54^. Groundwater input has been shown to correlate well with conductivity, as water in contact with bedrock is likely to become enriched in ions and minerals. The positive relationship between CO_2_ saturation and conductivity may thus indicate lateral input of CO_2_^55^, in particular in the studied subarctic and arctic systems. Further, lake pH may regulate the CO_2_ concentration with a higher proportion of the DIC staying as free CO_2_ at low pH values while at higher pH values, more CO_2_ enters the carbonate cycle, forming carbonate and bicarbonate. Boreal lakes are low in alkalinity (Fig. S1) and sensitive to changes in pH while Arctic systems due to glacial influence are well buffered. Therefore, besides serving as a substratum for heterotrophic bacteria, enhanced input of humic acids may result in a decline in pH and thus an additional increase in CO_2_ saturation in boreal lakes^56^ but hardly in well buffered arctic lakes.

The corresponding negative relationship between DOC concentration and O_2_ saturation suggests enhanced microbial O_2_ consumption, which may go along with a decrease in primary production when DOC inputs and thus light attenuation increases^21,57^. The net heterotrophy that is prevailing in boreal DOC-rich lakes is attributed both to reduced photosynthesis in the water column and enhanced microbial respiration^58^, implying that the degree of net heterotrophy increases with increasing DOC concentration in the boreal lakes.

### The role of nutrients

Phosphorus is the major limiting nutrient of bacterioplankton and has been previously shown to be a strong driver of lake CO_2_ supersaturation^13,59,60^. The influence of TP on the CO_2_:O_2_ ratio in the boreal lakes was positive up to about 15 µg DOC l^−1^, above which the effect declined and became negative. However, there were only few observations with high TP concentrations resulting in wide confidence interval in the second half of the curve (Fig. 3a). The ratio of CO_2_:O_2_ could also be affected by the bacterial carbon use efficiency which depends on the nutrient to C ratio of the substrate with high ratios allowing to allocate more C to growth while with low ratios, bacteria may dispose excess C as enhanced respiration^61,62^. Accordingly, CO_2_ saturation was negatively related to the TP:DOC ratio in boreal lakes (Fig. S4), suggesting enhanced rates of CO_2_ production in lakes where DOC concentrations were high in relation to TP concentrations.

Primary production in boreal lakes has been shown to be strongly affected by nitrogen availability in areas receiving low input of N^63^ and the positive effect of TN on O_2_ saturation likely reflects increased primary production with increased TN concentrations.

### Different dynamics in high-latitude lakes

A main result of our study was the difference between boreal and sub-Arctic and high-Arctic catchments in terms of CO_2_ and thus CO_2_:O_2_-ratio. In contrast to the boreal lakes, DOC concentration did not appear to drive the CO_2_:O_2_ ratio in Arctic lakes, and there was no significant trend in CO_2_ saturation in response to DOC concentrations. This was consistent both for sub-Arctic and high-Arctic sites despite the difference in catchment characteristics of the systems (birch forest/bog vs barren or glacier dominated catchments), and is in support of a recent comparison between boreal (forested) and Arctic sites^41^. The majority of the studied Arctic lakes were saturated or oversaturated with both O_2_ and CO_2_ without any relation between O_2_ and CO_2_ saturation level. These lakes are generally shallow and low in pelagic productivity, but may have a vigorous benthic production^64^. Also, the high-Arctic shallow sites are continuously wind-mixed, which could override internal metabolic drivers of CO_2_ and O_2_. In the cases of oversaturation, this can be attributed to the high contribution from dense mats of mosses and benthic algae in these localities. Hence, O_2_ saturation is most likely a result of both wind-induced mixing with support from benthic primary production in the Arctic sites.

Among the significant predictors of the CO_2_:O_2_ ratio in the Arctic, conductivity was clearly predominant (Table 2; Fig. 3b,c). This suggests that the majority of lake CO_2_ in the Arctic entered from the surrounding mineral soils^65^ and was not produced through in-lake DOC mineralization. For some high-Arctic sites, conductivity can also be coupled to their proximity to the sea. The lakes closest to the sea are also the most productive lakes since they are influenced by vegetation and most frequently visited by birds, yielding enhanced DOC and nutrients^43^.

While CO_2_ saturation increased with conductivity, we observed no correlation between conductivity and DOC concentration in neither the sub-Arctic nor the high-Arctic. Instead, conductivity was closely correlated with DIC concentrations and total alkalinity in both Arctic regions. In the sub-Arctic, CO_2_ saturation spanned from undersaturated to supersaturated. Saturation level was closely related to conductivity and thus also to total alkalinity. Alkalinity in turn could be coupled to bedrock, indicating weathering to be a source of lake DIC. While weathering of Si-holding rocks typically consumes CO_2_ via the Urey-reaction, others can generate DIC, e.g. via pyrite oxidation^66^. Likewise, the relatively high alkalinity together with the lime-rich bedrock suggests carbonate weathering to be a DIC source also in the high-Arctic lakes^41,67^.

In the high-Arctic lakes, O_2_ saturation correlated negatively with conductivity. While all lakes were supersaturated with O_2_, there was more variation in CO_2_ saturation with 7 out of 22 lakes being undersaturated or close to saturation and the rest supersaturated. The seven CO_2_ undersaturated lakes were all high in DOC and TN and highly influenced by birds. Many high latitude lakes are naturally poor in nutrients and although there was no significant relation between TN and O_2_ saturation, additions of N (and P) via birds may stimulate primary production, not the least of benthic autotrophs, with a concomitant drawdown of CO_2_. As bird feces also provide a source of organic C to lakes (geese feces typically has concentrations of 450, 11 and ca 3.2 mg per g dry weight for C, N and P respectively), and goose impacted lakes generally also have the highest levels of DOC^68^, also the heterotrophic bacterial production could to some extent be stimulated, yet we do not know how much of the fecal C eventually becomes accessible to pelagic bacteria as DOC.

The sub-Arctic lakes were all close to O_2_ saturation with little variation among lakes (95–99%), meaning that the variability in CO_2_:O_2_ ratio was primarily determined by CO_2_. Several studies suggest that Arctic lakes are net heterotrophic, as they are net emitters of CO_2_ to the atmosphere^2,69^. The majority of Arctic lakes in our study was indeed supersaturated with CO_2_ and net emitters of CO_2_ at the time of sampling. However, the uncoupling of CO_2_:O_2_ ratio from DOC and of CO_2_ and O_2_ saturation indicate decoupling of CO_2_ and O_2_ production. This decoupling could be the result of groundwater or glacial water CO_2_ inputs, or benthic primary production in surface sediments, while heterotrophic production dominates in deeper sediment layers. The high O_2_ saturation levels together with a dominance of allochthonous DIC suggest net system autotrophy rather than heterotrophy in these arctic lakes if only the autochthonous balance is considered. CO_2_ may also be produced from organic C mobilized by permafrost thaw, however, permafrost in the Scandinavian Arctic is generally only found in restricted palsas or in mountainous areas. Although allochthonous DIC may govern the CO_2_ in the arctic lakes in this study, heterotrophic mineralization of DOC may thus have an impact on CO_2_ saturation in arctic lakes affected by permafrost thawing.

### Conclusion

Based on a large number of (mostly) boreal lakes, Larsen et al.^38^ claimed DOC to be a universal predictor of lake pCO_2_, while groundwater influx was a minor contributor. However, including also sub- and high-Arctic lakes, we found a clear distinction in drivers of CO_2_ saturation along a latitudinal gradient. The boreal lakes followed the expected pattern with both CO_2_ and O_2_ saturation being largely dependent on DOC concentrations and relating negatively to each other, suggesting enhanced net heterotrophy with increased DOC inputs. In the Arctic lakes, despite the differences between sub-Arctic and high-Arctic sites, there was no correlation between DOC and CO_2_, yet these sites were also to a large degree supersaturated with CO_2_ and thus could be considered net heterotrophic. However, most of these lakes were also saturated or supersaturated with O_2_, indicative of low respiratory activity in agreement with generally nutrient-poor conditions and low levels of primary production. This is supported by the positive correlation between CO_2_:O_2_ ratio and conductivity, while the influence of DOC concentration was weak or non-significant. This may suggest that the major share of CO_2_ in these lakes is of allochthonous origin, likely from organic carbon mineralization and carbonate weathering in the catchment soils, entering via groundwater flow^41,55^. This points to fundamentally different drivers of CO_2_ and O_2_ concentrations in boreal and Arctic lakes due to differences in vegetation, landscape structure, hydrology and lake bathymetry.

## Supplementary Information

Supplementary Figures.

## Data Availability

All data will be made available upon request to Lina Allesson, or if demanded, stored in a UiO repository (Git-HUB).
