# Supplementary material for: Drivers and variability of CO2:O2 saturation along a gradient from boreal to Arctic lakes
Source: Sci Rep. 2022 Nov 8;12:18989. doi: 10.1038/s41598-022-23705-9 (PMC9643447; doi:10.1038/s41598-022-23705-9)
Supplement: Supplementary file 1 — Supplementary Figures. [file 41598_2022_23705_MOESM1_ESM.docx]

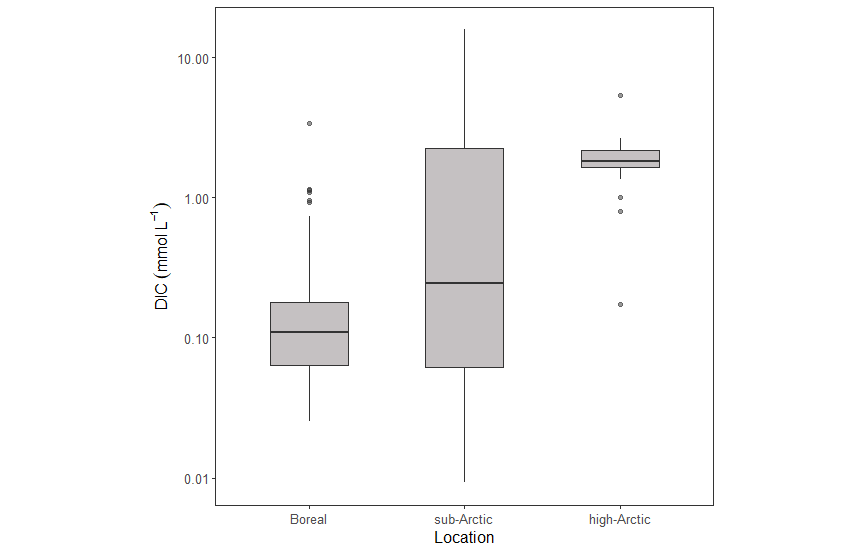


Fig. S1 Boxplot of total DIC concentration in the three regions. In the Boreal part, where granitic and metamorphic bedrock dominates, the alkalinity is generally low. The wide variability in alkalinity in the sub-Arctic is a result of the variability in bedrock from granitic to sedimentary (slate). In the high-Arctic, the majority of the lakes are situated on limestone bedrock giving elevated alkalinity.


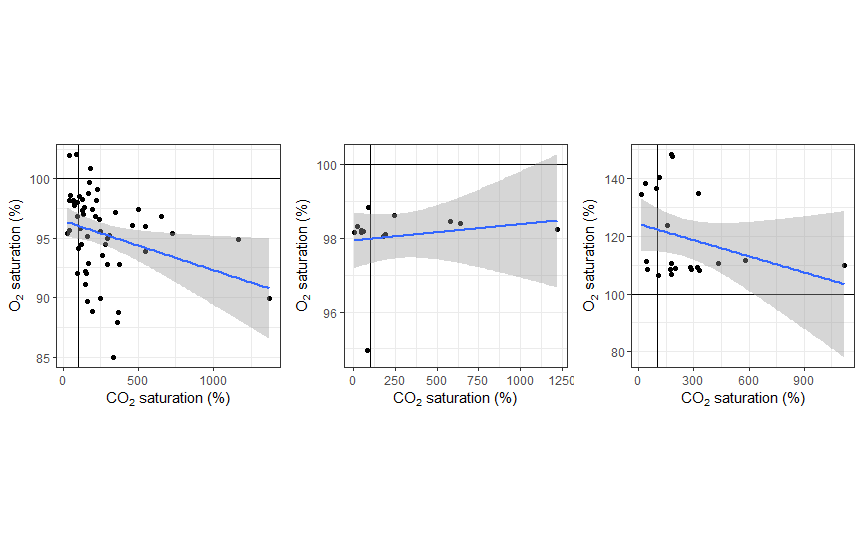


Fig. S2 Scatterplots of O_2_ saturation vs. CO_2_ saturation. From left to right Boreal lakes, sub-Arctic lakes, high-Arctic lakes. Although quite some scattering, there was a weak but significant negative relation between O_2_ saturation and CO_2_ saturation in the boreal lakes (ρ = -0.42). The relation was stronger in lakes with CO_2_ saturation below 500 %. In the northern lakes (sub-Arctic and high-Arctic) where all lakes were saturated or subersaturated with O_2_, there was no significant relation between O_2_ and CO_2_ saturation. Note differences in axes, lines indicate 100 % saturation.


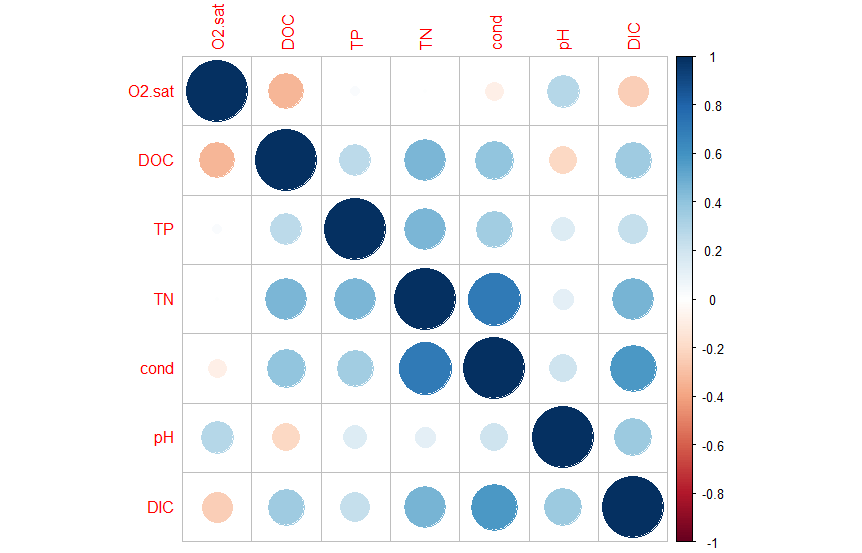


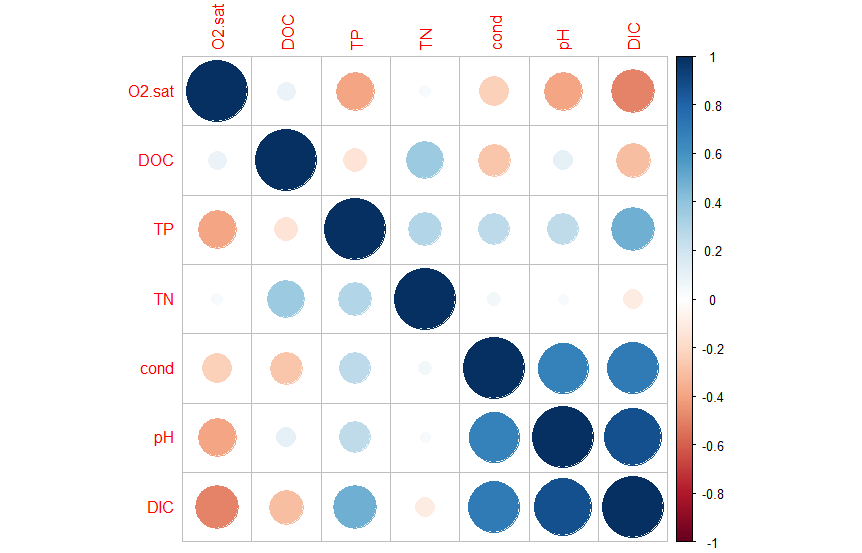


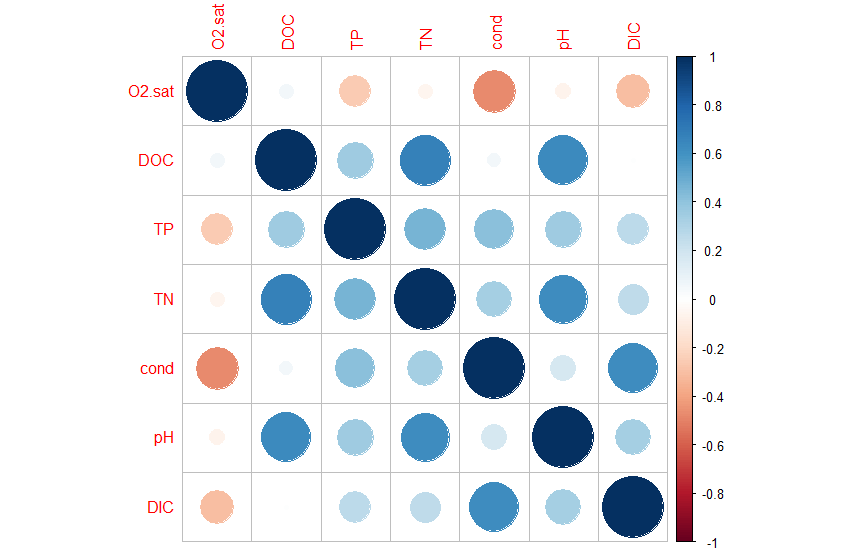


Fig. S3 Spearsman’s correlation matrices of lake chemistry variables and O_2_ saturation. Top to bottom: Boreal lakes, sub-Arctic lakes, high-Arctic lakes.


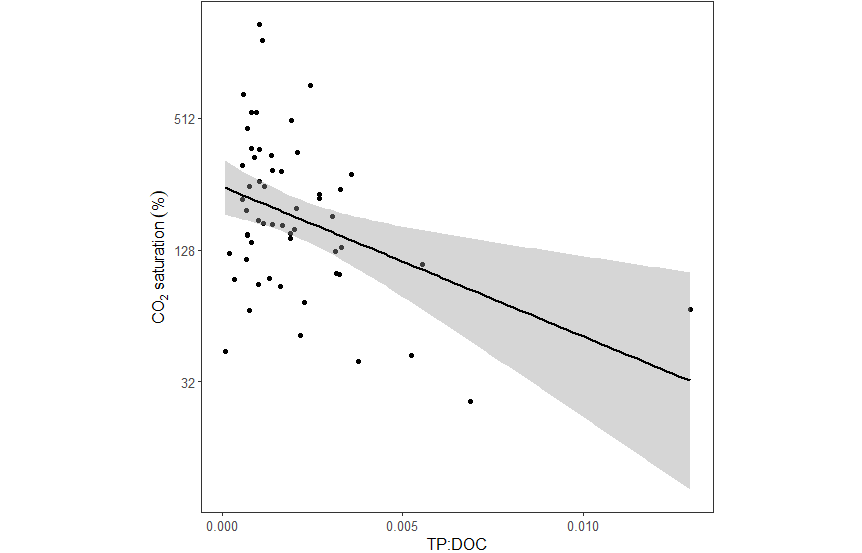


Fig. S4 CO_2_ saturation vs TP:DOC ratio in the boreal lakes. The CO2 saturation level decreases as TP:DOC increases.
